# Preparation and Evaluation of Nanofibrous Hydroxypropyl Cellulose and β-Cyclodextrin Polyurethane Composite Mats

**DOI:** 10.3390/nano10040754

**Published:** 2020-04-15

**Authors:** Luiza Madalina Gradinaru, Mihaela Barbalata-Mandru, Mioara Drobota, Magdalena Aflori, Maria Spiridon, Gratiela Gradisteanu Pircalabioru, Coralia Bleotu, Maria Butnaru, Stelian Vlad

**Affiliations:** 1 “P. Poni” Institute of Macromolecular Chemistry, 41A Grigore Ghica Voda Alley, 700487 Iasi, Romania; mihaelamandru84@gmail.com (M.B.-M.); miamiara@icmpp.ro (M.D.); maflori@icmpp.ro (M.A.); dana_spiridon_is@yahoo.com (M.S.); mariabutnaru@yahoo.com (M.B.); 2 Sanimed International Impex S.R.L, 70F Bucuresti—Măgurele, 051434 Bucuresti, Romania; gratiela87@gmail.com (G.G.P.); cbleotu@yahoo.com (C.B.); 3 “Stefan S Nicolau” Institute of Virology, 285 Mihai Bravu, 030304 Bucuresti, Romania; 4 Faculty of Medical Bioengineering, “Gr. T. Popa” University of Medicine and Pharmacy, 700115 Iasi, Romania

**Keywords:** biocompatibility, cytotoxicity, electrospinning, nanofibers, polyurethane composites

## Abstract

A series of nanofibrous composite mats based on polyurethane urea siloxane (PUUS), hydroxypropyl cellulose (HPC) and β-cyclodextrin (β-CD) was prepared using electrospinning technique. PUUS was synthesized by two steps solution polymerization procedure from polytetramethylene ether glycol (PTMEG), dimethylol propionic acid (DMPA), 4,4′-diphenylmethane diisocyanate (MDI) and 1,3-bis-(3-aminopropyl) tetramethyldisiloxane (BATD) as chain extender. Then, the composites were prepared by blending PUUS with HPC or βCD in a ratio of 9:1 (w/w), in 15% dimethylformamide (DMF). The PUUS and PUUS based composite solutions were used for preparation of nanofibrous mats. In order to identify the potential applications, different techniques were used to evaluate the chemical structure (Fourier transform infrared-attenuated total reflectance spectroscopy—FTIR-ATR), morphological structure (Scanning electron microscopy—SEM and Atomic force microscopy—AFM), surface properties (contact angle, dynamic vapors sorption—DVS), mechanical characteristics (tensile tests), thermal (differential scanning calorimetry—DSC) and some preliminary tests for biocompatibility and microbial adhesion.

## 1. Introduction

Electrospinning, an electrostatic fiber manufacturing technique, has shown more interest and attention in recent years due to its versatility and potential to be applied in various fields. This is a relatively simple and convenient technique to produce nanofibers from a wide variety of natural or synthetic polymers, or their blends. Electrospinning is a unique technology that can produce non-woven fibrous materials with fiber diameters ranging from nanometers to microns. Electrospinning process can be controlled by many parameters, such as solution parameters (concentration, viscosity, conductivity, etc.), processing parameters (applied voltage, flow rate, etc.) or ambient parameters (temperature, humidity) [1]. Electrospun nanofibers still represent a relatively new class of advanced biomaterials. In the last decade, many potential applications are being explored in various fields, due to their large surface area to volume ratio, unique fibrous porosity architecture, interconnected pore structure and malleability to prepare a wide variety of sizes and shapes [2]. Electrospun matrices can simulate the structure of the extracellular matrix, possess good biocompatibility and favors cells colonization. Thus, electrospun nanofibers are broadly investigated in biomedical applications, as tissue engineering scaffolds [3,4], wound dressing [5,6], drug delivery [6,7], enzyme immobilization [8,9], etc. 

Nowadays, a large variety of polymers can be electrospun and the nanofibers from these polymer solutions have been used in various applications. Among them, polyurethanes are an important and versatile class of polymers that have found widespread applications in biomedical field [10]. Generally, polyurethane based materials are widely used in coatings [11], paints [12], foams [13], elastomers [14,15,16], adhesives [17] and medical devices [18,19,20]. Thereby, polyurethane based materials have been the subject of several studies aimed to design advanced materials with high performances [21,22,23]. In addition to polyurethanes, polyurethane nanofibers are also of great interests to researchers and have been investigated by numerous research groups [24,25]. By combining the properties of polyurethanes and electrospinning, there is the possibility to obtain scaffolds that can mimic the native extracellular matrix of biological tissue. Therefore, due to the large surface area and porous network structure, the electrospun polyurethane nanofibers can be used effectively for many biomedical applications such as in wound dressing preparation [26,27], drug delivery systems [28], tissue engineering [29,30], etc.

Fabrication of composites at nanoscale level is a facile method to develop or modify structural and functional heterogeneous materials. Mechanical incorporation of some filler into polymeric matrices is a promising approach to fabricate high performance nanocomposites. Composite nanofiber mats are produced by the combination of synthetic and/or natural polymers that possess advantages of each polymer to overcome their limitations and aiming specific biomedical applications. Hydroxypropyl cellulose (HPC) is a non-ionic and hydrophilic cellulose derivate which is used in different applications in biomedical, pharmaceuticals, food additive, etc. In biomedical applications it is used in the field of ophthalmology as a lubricant for artificial eyes [31], in drug delivery, tissue engineering, wound healing, etc. [32]. β-Cyclodextrin (β-CD), another biobased monomer with a lipophilic cavity and a hydrophilic exterior, has attracted also attention due to its accessibility, lowest cost, environmentally friendly, excellent biocompatibility and ability to form inclusion complexes [33]. This natural product, which is obtained through enzymatic conversion from starch, is extensively used in separation and purification [34,35].

Although literature shows a high number of studies [24,25,26,27,28,29,30], the possibility for combining the properties of the aforementioned compounds with the purpose of creating a novel or improved generation of fibrous scaffolds is still attractive. To realize this, it is important to study the interaction between the structures used to obtain the polymeric matrices, in order to prepare further new scaffolds for biomedical applications. This is the first and an important step in the preparation of scaffolds with enhanced properties. Thus, the aim of this work was to prepare polyurethane based composite mats by electrospinning technique and to understand the influence of HPC and BCD structures on the structural, morphological and biological properties. To achieve this purpose, it was first synthesized a polyetherurethane urea siloxane (PUUS) by two steps solution polymerization procedure. For this synthesis, a soft segment polytetramethylene ether glycol (PTMEG) and dimethylol propionic acid (DMPA) was used, and for the hard segment 4,4′-diphenylmethane diisocyanate (MDI) and 1,3-bis-(3-aminopropyl) tetramethyldisiloxane (BATD) were used. For composites fabrication, solutions of PUUS blended with HPC or β-CD in a ratio of 9:1 (w/w) were prepared in 15% DMF. The PUUS and PUUS based composite solutions were used for preparation of nanofibrous mats. The prepared nanofiber mats were investigated using a series of characterization methods, including Fourier Transform infrared-attenuated total reflectance spectroscopy (FTIR-ATR), scanning electron microscopy (SEM), atomic force microscopy (AFM) and dynamic vapor sorption (DVS). In addition, static contact angle, mechanical and thermal (DSC) properties have also been investigated and correlated. Preliminary in vitro biocompatibility and microbial adhesion tests were also performed. This study indicated that the developed polyurethane based nanofibrous composite mats with enhanced physico-chemical properties and biocompatibility showed great promise and potential for many biomedical applications, such as tissue engineering, wound dressing, immobilized enzymes and controlled drug delivery.

## 2. Materials and Methods 

### 2.1. Materials

Polytetramethylene ether glycol (PTMEG-Terathane^®^) average Mn ~2000 g mol^−1^ was kindly offered by Invista BV Netherland, 4,4′-diphenylmethane diisocyanate (MDI) was purchased from Fluka (Steinheim, Germany), and was fresh distilled prior to use. Dimethylolpropionic acid (DMPA) purum, dibutyltin dilaurat (DBTL) and dimethylformamide (DMF), were also obtained from Fluka (Steinheim, Germany). Commercial DMF was dried over anhydrous K_2_CO_3_, and then was distilled from calcium hydride (CaH_2_) and kept over 4 Å molecular sieves. 1,3-bis-(3-aminopropyl) tetramethyldisiloxane (BATD) was purchased from Alfa Aesar GmbH & Co KG, Karlsruhe, Germany. Hydroxypropyl cellulose (HPC), Mn ~10,000, powder, 20 mesh particle size and β-cyclodextrin (β-CD) were purchased from Sigma-Aldrich (Steinheim, Germany). The other chemicals were used as received without further purification.

### 2.2. Synthesis of PUUS

PUUS was synthetized following the same procedure previously reported [36]. Briefly, into reaction vessel, PTMEG and DMPA were dehydrated under vacuum, temperature and stirring. In the next step, under normal conditions, DMF as solvent was added. After complete dissolution, MDI and DBTL were added, and a homogenous prepolymer was obtained. After two dilution steps, the prepolymer was cooled, and then extended with a solution of BATD in DMF. Then, the temperature was increased gradually until the polymer solution becomes clear. Afterwards, the reaction medium was precipitated in worm distilled water and then allowed to cool. The polymer was washed a few times with distilled water to remove the solvent. The molar ratio of the reactants MDI:PTMEG:DMPA:BATD was 3:1:1:1. The obtained polymer was dried under vacuum for a few days. 

### 2.3. Preparation of Spinning Solutions

Three different spinning solutions of PUUS, PUUS-HPC and PUUS-βCD were prepared. Thus, 15% (w/w) of PUUS was first dissolved in DMF using a magnetic stirrer for 6 h. Then, certain amount of HPC or β-CD was added and vigorously stirred. The ratio between PUUS and fillers (HPC or β-CD) was 9:1 (w/w). All the solutions were freshly made before electrospinning experiments. 

### 2.4. Electrospinning Process

**Figure 1:**
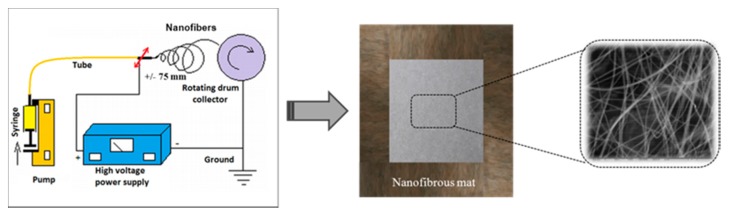
Schematic illustration of the experimental electrospinning procedure and preparation of polyurethane based nanofibrous composite mats.

The prepared solutions of PUUS, PUUS-HPC and PUUS-βCD in DMF were used for antigravity electrospinning experiment. The apparatus contains four elementary components: needle, syringe pump, high voltage power supply and collector. Figure 1 illustrates the basic components required to perform electrospinning as mentioned above. A 10 mL glass syringe is filled with polymer solution which it is driven through a needle into an electric field using a programmable syringe pump (SEP-21S Plus, Vilnius, Lithuania) at a rate of 0.5 mL h^−1^, for 10 h. The pump allows the solution to be introduced into system at a precisely controlled rate. The metallic blunt needle (Type 22G 11/2; inner diameter = 0.413 mm) is connected to a high voltage power supply that is capable of generating voltage between 0 and 30 kV. In order to create an electric field, the system must contain, along with the charged needle, a grounded drum. The conductive drum completes a circuit and allows a strong electric field to be created between the needle and collector. The glass syringe is connected with the blunt needle through a tube with 1.6 mm inner diameter (Tygon^®^ 2375 Ultra Chemical Resistant Tubing). The positive lead from a high voltage supply was connected via an alligator clip to the external surface of the needle. The blunt needle is fitted on the oscillating device in the horizontal plane, aided by computer which can adjust the distance and frequency oscillation (in this case the distance was of +/− 70 mm, frequency of the complete oscillation of 2.8 s and delay on heads of 0.1 s). The aluminum drum collector (diameter 60 mm, length 150 mm and 180 rpm) was placed at 80 mm from the tip of the needle. The voltage was kept at 15 kV, using a High Voltage Power apparatus, type: HPC 140–35,000 (0–35,000 V; 0–4 mA) FUG Elektronik GmbH Rosenheim—Germany. Antigravity electrospinning procedure was conducted at ambient conditions (typically 25 °C, relative humidity: 65%). An overview of the experimental electrospinning conditions for the preparation of polyurethane based nanofibrous composite mats is shown in Table 1. After separation from drum collector, the finished polyurethane based nanofibrous composite mats were washed in distilled water for 24 h and dried in vacuum oven at 40 °C for 48 h. 

### 2.5. Methods

#### 2.5.1. Fourier Transform Infrared-Attenuated Total Reflectance Spectroscopy (FTIR-ATR)

The infrared spectra were obtained using a Bruker LUMOS—FTIR Microscope (Bruker Optik GmbH, Ettlingen, Germany) with ATR reflection module (attenuated total reflection) and a diamond crystal, at single reflection of 45° angle equipped with OPUS 8 software (Version 8, Ettlingen, Germany) for spectral processing. The mat surfaces were scanned in the 600–4000 cm^−1^ range. The all spectra were collected by cumulating 64 scans at a resolution of 2 cm^−1^. The spectra were recorded at a constant temperature of 25 °C.

#### 2.5.2. Scanning Electron Microscopy (SEM)

Surface morphology was examined using a FEI Quanta 200 scanning electron microscope (FEI Company, Brno, Czech Republic) equipped with EDAX Si (Li) X-ray detector and Gatan Alto Cyro stage, in a high-vacuum microscope chamber at an accelerating voltage of 20 kV. Mat samples were mounted on graphite supports and observed under different degrees of magnifications. For each mat samples, several measurements were performed and analyzed.

#### 2.5.3. Atomic Force Microscopy (AFM)

Atomic force microscopy (XE-100, Park Systems Corp, Mannheim, Germany) was used to study the surface structure of the mat samples by measuring the force between atoms, working under standard ambient environment. Surfaces of 5 μm × 5 μm of the mat samples were scanned, using a cantilever type Olympus AC240TS (*f* = 70 kHz; *k* = 2 N m^−1^) by non-contact measures surface topography, in the relatively larger distance between the tip and the sample surface. A charge coupled device (CCD) camera is aligned directly on the cantilever in order to provide high quality of the images. Differences in the surface morphology can be expressed in terms of various roughness parameters, such as: Arithmetic roughness (*R*_a_) which represents the arithmetic mean of the height of peaks and depth of the valleys from a mean line, *R*_ku_ (kurtosis)—characterizes the flatness of the surface distribution, *R*_sk_ (skewness)—characterizes the asymmetry of the surface distribution or root-mean-square roughness (*R*_q_) [37]. These parameters were determined from five separate images of different locations of the surfaces of each mat samples.

#### 2.5.4. Stress–Strain Measurements

Stress–strain measurements were performed on Instron apparatus (INSTRON model 3365; Universal Testing Machine, INSTRON, Norwood, MA, USA), with a load cell of 500 N, on dumbbell-shaped cut mat samples of 50 mm total length, 8.5 mm gripped width and 4 mm active area width. The thickness was measured for each cut mat with a digital micrometer. The tests were performed at an extension rate of 30 mm min^−1^ at room temperature (20–22 °C). For statistical significance 3 specimens of each sample were tested and average values of strength and elongation at break were determined.

#### 2.5.5. Static Contact Angles Measurements

To monitor the wettability of the mat samples, the static contact angles were measured by sessile drop technique at room temperature, using CAM 101 Optical Contact Angle by KSV Instruments Ltd., Helsinki, Finland. Images were recorded with a special optical system equipped with a CCD camera connected to a computer. The solvent used was distilled water. A drop of liquid (~1 μL) was placed, with a Hamilton syringe, on a specially prepared plate of substratum and the image was immediately sent via the CCD camera to the computer for analysis. All the measurements were done in triplicate and the results were recorded as mean ± standard deviation. The angle formed between the liquid/solid interface and the liquid/vapor interface is the contact angle. Temperature and moisture were constant during the experiment (25 °C and 65% respectively).

#### 2.5.6. Dynamic Vapors Sorption (DVS)

Dynamic vapors sorption capacity of the mat samples was measured in dynamic regime by using an IGAsorp apparatus (a fully automated gravimetric analyzer, supplied by Hiden Analytical, Warrington, UK). This apparatus is used to study water sorption at atmospheric pressure by passing a humidified stream of gas over the sample, and can be applied to a wide range of studies. The IGAsorp is a standard sorption equipment, which has a sensitive microbalance (resolution 1 mg and capacity 200 mg), which continuously registers the weight of the sample together with the temperature and relative humidity around the sample. 

#### 2.5.7. Differential Scanning Calorimetry Analysis (DSC) 

The thermal properties of the prepared mat samples were investigated by differential scanning calorimetry (DSC 200 F3 Maia, Netzsch-Geratebau GmbH, Selb Germany). The DSC analysis was performed by heating a mass of 10 mg of each sample in pressed and pierced aluminum crucibles at a heating rate of 10 °C min^−1^, under N_2_ atmosphere (50 mL min^−1^). The baseline was obtained by scanning the temperature domain of the experiments with an empty pan. Temperature and sensitivity calibrations were performed with five different metals at various heating rates according to standard procedures. For each sample a single measurement was performed. 

#### 2.5.8. Biological Study

A first preliminary cytotoxicity tests were performed using human dermal fibroblasts (HDF) cells and yellow 3-(4,5-dimethylthiazol-2-yl)-2,5-diphenyl tetrazolium bromide (MTT) assay. The cells were given with courtesy by the Head of Tissue Engineering and Regenerative Medicine Laboratory of Biomedical Department, University of Medicine and Pharmacy Grigore T Popa from Iasi, Romania. The primary HDF is a purchased cell line ATCC^®^ PCS-201-012, cultured, passed and stored at ultralow temperature. Briefly, the routine cell culture protocol for HDF cell line after thawing consisted of cell washing by centrifugation at 350G for 10 min in Dulbecco’s Modified Eagle Medium/F-12 medium (DMEM/F-12, Gibco, ThermoFisher Scientific) culture medium supplemented with 10% fetal bovine serum (FBS) and 1% antibiotic mixture of penicillin, streptomycin and neomycin (PSN). The cell pallet was resuspended in fresh media and the resulted cell suspension was seeded on the surface of 75 cm^2^ culture flask for growing until subconfluency of cultured cell was reached (about 3 days of culture). HDF subconfluent cell layer was harvested using trypsin/EDTA (0.25%/0.02%) solution for 2 min at 37 °C, centrifuged at 350G for 10 min and resuspended in fresh DMEM/F-12 culture medium to achieve a suspension of 4 × 10^4^ cells/mL used for subsequent seeding of 24-well culture plates in MTT cytotoxicity assay. Before cell proliferation assay, mat samples were sterilized for 15 min in 70% v/v ethyl alcohol solution, washed in sterile phosphate buffer (PBS—Sigma) and then incubated in DMEM media at 37 °C and 5% CO_2_ atmosphere for 48 h. After that, the mats were transferred on the 24-well cell culture plate. One sample was used for each plate well in the triplicate mode. Upon the samples, an initial concentration of 4 × 10^4^ HDF cells/well was added and incubated for 24, 48 and 72 h. After each desired incubation time, culture media from the wells was removed and 0.5 mL of MTT solution in DMEM was added to each (control and sample) well. The final concentration of MTT/well was 0.25 mg mL^−1^. The culture plate was incubated for 3 h at 37 °C in dark conditions. After incubation, as a result of viable cells activity, the yellow MTT compound was reduced in a violet colored product (formazan), which was solubilized by adding 0.5 mL/wall of 2-propanol. The absorbance of the colored solution obtained was quantified by the spectrophotometer measuring at *λ* = 570 nm. The cell viability was normalized to that of fibroblasts cultured in the media with negative control (without material).

Another cytotoxicity test was performed by using human epidermoid cells (HEp2) (American Cell Type Collection [ATCC] CCL-23, VA, USA), cells which are widely used for the determination of the cytotoxic effects of different materials. Briefly, the HEp-2 cell line was cultivated in Dulbecco’s Modified Eagle Medium (DMEM) supplemented with 10% heat-inactivated bovine serum and penicillin/streptomycin at 37 °C with 5% CO_2_. The adherent cells were detached, centrifuged, suspended in fresh medium, counted by trypan blue exclusion and adjusted to 1 × 10^5^ cells mL^−1^. Cells were cultivated for 24 h at 37 °C, in 5% CO_2_. Then, the sterilized sample mats were placed in a 24-well plate and subsequently 1 × 10^5^ HEp2 cells were added to each well containing mats. After 24 h, the cell morphology was observed using contrast microscopy (AxioSCope D1, Zeiss). 

To analyze the effect harbored by the samples on the cell cycle, the cells were trypsinized at 24 h, washed in PBS and fixed in ice cold 70% ethanol. Subsequently, cells were treated with RNAse (1 mg mL^−1^) at 37 °C for 30 min and stained with propidium iodide (100 μg mL^−1^). The samples evaluation was realized using a flow cytometer Beckman Coulter XLM (Winooski, VT, USA) and a Flow Jo7 software (version 7.0, Partek Inc., St. Louis, MO, USA).

The study of biomaterial resistance to microbial adhesion with microbial biofilm formation at the surface of composites mats was evaluated using an earlier reported protocol [38]. The mat samples were cut into squares (1 × 1 cm), placed in 24-well plates, sterilized with 70% ethanol (1) and washed with PBS (3 times). The mat samples were then incubated with 1 mL of bacterial suspension of different strains of *Staphylococcus aureus* and *Pseudomonas aeruginosa*, according to 0.5 McFarland turbidity standards. The plates were placed in the incubator at 37 °C for 2 h. Afterwards, the mats were removed from the 24-well plates using a sterile forceps and washed three times with PBS to ensure removal of the non-adherent bacteria. The mat samples were then placed in tubes with 1 mL of PBS and vortexed for 120 s to remove all solutions from the adhering bacteria. Then, the solution was serially diluted in PBS, grown on nutrient agar and the number of colony forming units per mL (CFU/mL) was calculated. 

## 3. Results

The properties of the nanofibrous composite based on PUUS and PUUS blended with HPC and β-CD prepared by electrospinning technique, were investigated using a series of characterization methods, including Fourier transform infrared-attenuated total reflectance spectroscopy (FTIR-ATR), scanning electron microscopy (SEM), atomic force microscopy (AFM) and dynamic vapor sorption (DVS). In addition, static contact angle, mechanical and thermal (DSC) properties have also been investigated and correlated. Preliminary in vitro biocompatibility and microbial adhesion tests were also performed.

### 3.1. FTIR-ATR Characterization

**Figure 2:**
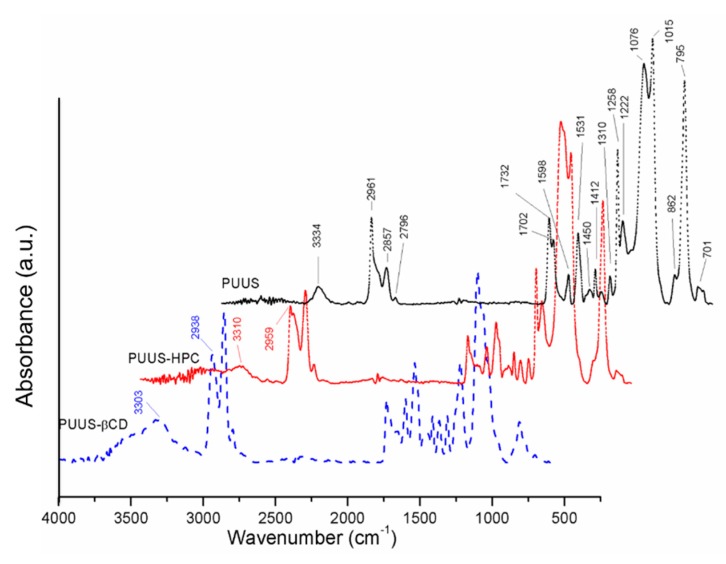
FTIR-ATR spectra of nanofibrous mat (polyurethane urea siloxane (PUUS)) and nanofibrous composite mats (PUUS-hydroxypropyl cellulose (HPC) and PUUS-β-cyclodextrin (βCD)).

The chemical structure of the PUUS was confirmed by FTIR-ATR. The formation of the polyurethane structure is confirmed by the disappearance of NCO peak at 2270–2250 cm^−1^ (Figure 2). This indicates that isocyanate groups have reacted with hydroxyl and amino groups to form urethane and urea bonds. Moreover, the absorption bands around 3334 cm^−1^ (N–H stretching), 1531 cm^−1^ (N–H bending), 1732 and 1702 cm^−1^ (free and bonded C=O stretching of urethane) and 1258–1222 cm^−1^ (stretching vibration of N–CO–O) confirm the formation of the urethane linkage [36,39]. The peaks at 2961, 2857 and 2795 cm^−1^ (–CH_2_ and –CH_3_ stretching vibration); 1598 cm^−1^ (C=C stretching vibration of MDI); 1450 and 1310 cm^−1^ (–CH_2_ and –CH_3_ deformation vibration); 1076 and 1015 cm^−1^ (C–O–C stretching vibration of PTMEG and Si–O–Si asymmetric stretching vibration); 862 and 795 cm^−1^ (Si–C stretching) can clearly be observed in the spectra [40].

By analyzing the corresponding spectra of nanofibrous composite mats (PUUS-HPC and PUUS-βCD), it was observed that the peak at 3334 cm^−1^ produced by stretching of the –NH and –OH groups in the spectrum of PUUS, becomes broader and shifts to smaller values at 3310 cm^−1^ (PUUS-HPC) and 3303 cm^−1^ (PUUS-βCD), respectively. The peak shift is due to the presence of hydrogen-bond structures in these blends [41]. Moreover, a new broad shoulder appears at around 3490 cm^−1^, characteristic to –OH groups from HPC and β-CD, respectively. The other characteristic vibration bands from HPC and β-CD are overlapping with those of PUUS, but the corresponding peaks are shifted to lower wavenumbers, due to the different inter- and intramolecular interactions. These results confirm that the HPC and β-CD have been successfully incorporated in the PUUS nanofibrous mats.

### 3.2. Scanning Electron Microscopy Analysis (SEM) 

**Figure 3:**
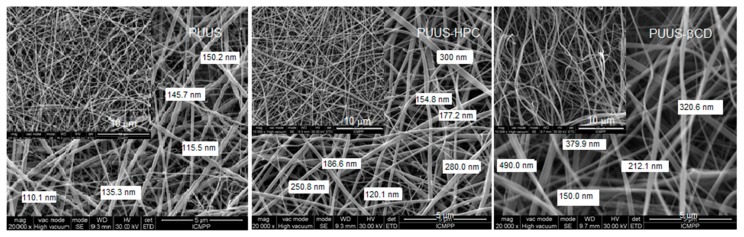
SEM images of nanofibrous mat (PUUS) and nanofibrous composite mats (PUUS-HPC and PUUS-βCD).

SEM is a useful method to evaluate the basic characteristics of prepared nanofibrous mats, such as fiber diameter or roughness. Moreover, it enables to reveal artifacts in the nanofibrous structures arising during electrospinning process. The surface morphology of the obtained nanofibrous composite mats based on PUUS, HPC and β-CD were characterized by SEM. The SEM images depicted in Figure 3 show that the obtained nanofibers were uniformly distributed, randomly oriented with the diameter at the nanoscale level ranged from 110 to 490 nm. In terms of fiber surface morphology, they are smooth, bead-free, without noticeable difference between the samples. However, a slight increase in fiber thickness is observed at the composite mats. This varied between 110–150 nm at PUUS, 120–300 nm at PUUS-HPC and 150–490 nm at PUUS-βCD, respectively.

### 3.3. Atomic Force Microscopy Analysis (AFM)

**Figure 4:**
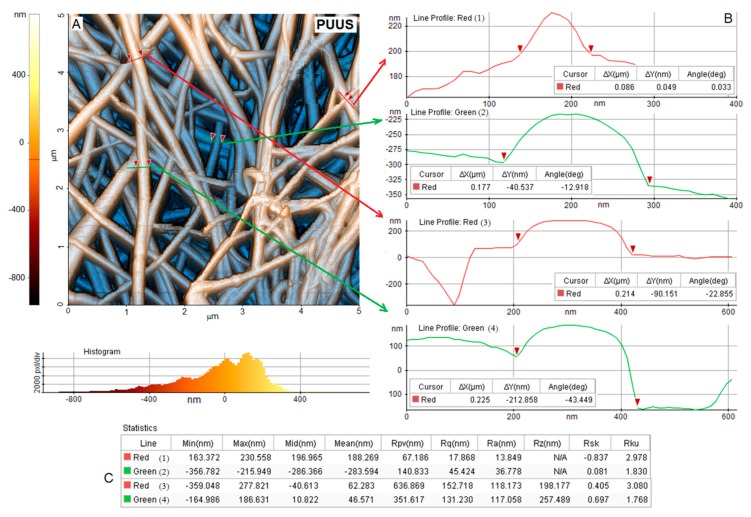
AFM morphology of PUUS nanofibrous mats: (**A**) 2D image of AFM analysis; (**B**) the diameters of four selected nanofibers (noted with red and green line); and (**C**) roughness values.

**Figure 5:**
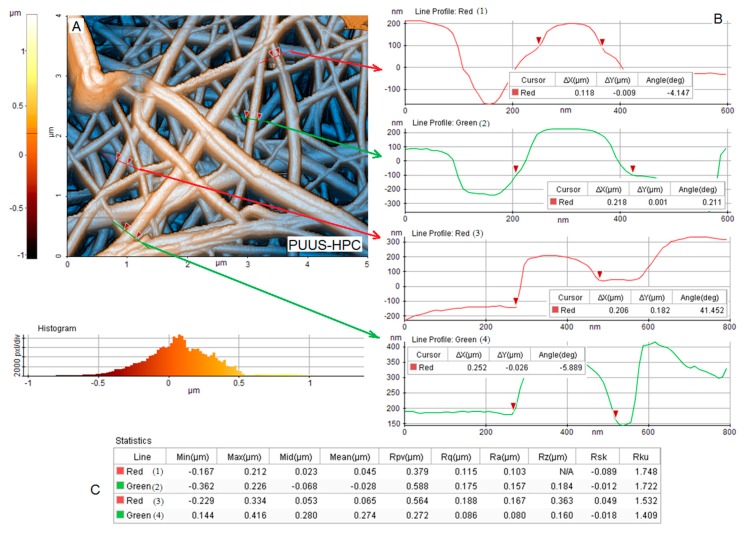
AFM morphology of PUUS-HPC nanofibrous composite mats: (**A**) 2D image of AFM analysis; (**B**) the diameters of four selected nanofibers (noted with red and green line); and (**C**) roughness values.

**Figure 6:**
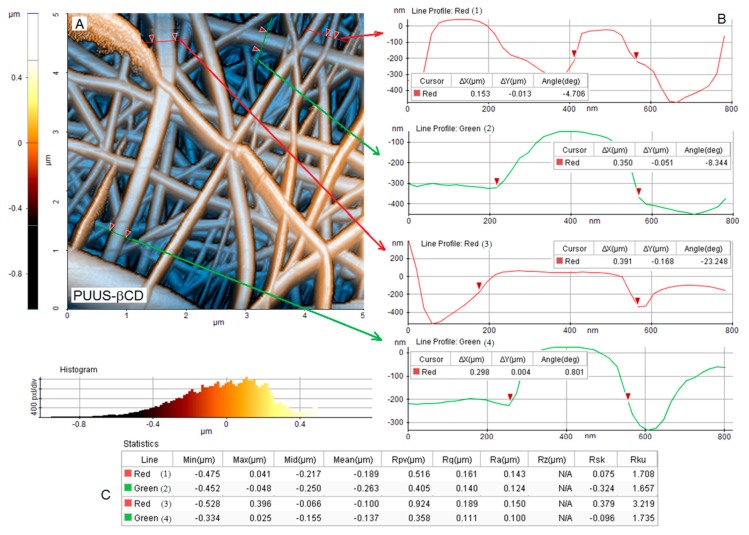
AFM morphology of PUUS-βCD nanofibrous composite mats: (**A**) 2D image of AFM analysis; (**B**) the diameters of four selected nanofibers (noted with red and green line); and (**C**) roughness values.

AFM is considered an extensively used technique for the characterization of nanofibrous materials, which give information about the topography, morphology and fiber distribution from the surface of the samples. Thus, the surface morphology of the nanofibrous mats was further performed using the atomic force microscopy, in non-contact mode. Figure 4, Figure 5 and Figure 6A show the 2D AFM-phase images of the obtained nanofibrous mats: PUUS (Figure 4A), PUUS-HPC (Figure 5A) and PUUS-βCD (Figure 6A), respectively. The obtained mats exhibited randomly oriented and interconnected fibrillar structures with relatively smooth surfaces. Some of the small fibers were bundled together leading to the formation of multi-fiber structures. In all cases, the prepared fibers led to the formation of interlaced structures responsible for the creation of relative regular networks. Moreover, these images illustrate that all the sample mats showed similar diameters of the fibers between 50 and 400 nm. Then, we choose certain fibers and we calculated their size. Thus, on the right side of these figures (noted with B) are illustrated the diameter profiles of the selected nanofibers. The fiber diameters of PUUS mats varied between 86 and 225 nm (Figure 4B), of PUUS-HPC are between 118 and 252 nm (Figure 5B) and of PUUS-βCD are between 153 and 391 nm (Figure 6B), respectively. Therefore, the same trend of fiber size increasing was observed at the composite mats, as in SEM images.

In order to investigate the surface roughness of nanofibrous mats, a narrow AFM scan range (5 × 5 μm) was adopted in this study. Two profiles were extracted for each area with several dimensional measurements. The roughness of the surfaces of the analyzed samples was determined from four measurements, selecting a representative domain for each specimen [42]. The measured roughness parameters are illustrated in the tables located at the bottom of each figure (noted with C). For example, the arithmetic roughness (*R*_a_) of the selected domain was between 13–118 nm for PUUS (Figure 4C), 80–167 nm for PUUS-HPC (Figure 5C) and 100–143 nm for PUUS-βCD (Figure 6C), respectively.

### 3.4. Mechanical Properties

**Figure 7:**
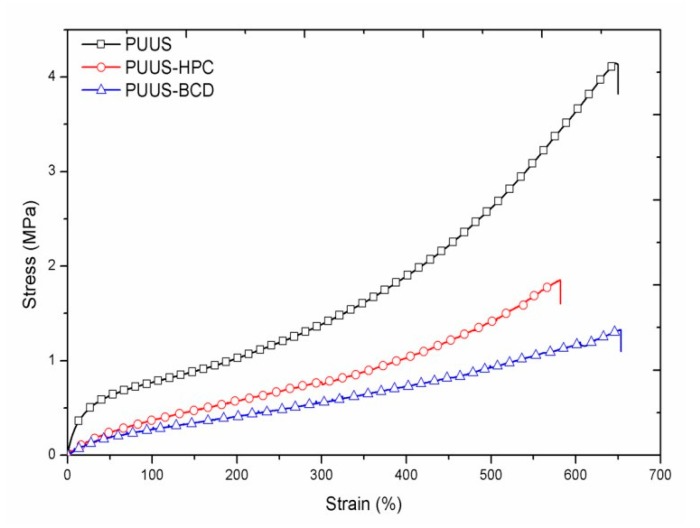
Stress–strain curves of the nanofibrous mats (PUUS) and nanofibrous composite mats (PUUS-HPC and PUUS-βCD).

The mechanical parameters are fundamentally important for the effective performance of the biomaterial in biological systems. Therefore, tensile testing measurements were performed and the typical stress–strain curves of the nanofibrous mats are shown in Figure 7. The addition of the natural components (HPC and β-CD) affects more the tensile strength of the nanofibrous mats and less their ultimate elongation. The tensile strength of the nanofibrous composite mats decreased from 11.57 MPa at PUUS to 4.80 and 4.16 MPa at PUUS-HPC and PUUS-βCD respectively, losing almost 30% of tensile strength. Thus, the composites require less force to break, acting as a plasticizer, which increases the free volume between the polymer chains and allows the chain segments to move and rotate more freely, leading also to a slight decrease of *T*g, as was observed in DSC analysis [43].

The parameters obtained from the stress–strain curves are summarized in Table 1. Tensile modulus or Young modulus was calculated from the stress–strain curve in the elastic domain and describes the elastic force of the sample that resist to deformation [44]. This modulus decreases from 3.90 MPa at PUUS to 0.87 and 0.73 MPa at PUUS-HPC and PUUS-βCD, respectively, meaning that the nanofibrous composite mats deform more easily than the pristine PUUS. This suggests an improvement of the elastomeric characteristics, which may be an advantage for some biomedical applications. For example, the biomaterials used in cardiac tissue engineering must have enough elasticity to support the cyclic loading of the scaffolds and to allow tissue relaxation at the end of contraction [45]. Toughness, the energy of the sample that can absorb before it breaks, decreases from 11.57 MPa at PUUS at around 4 MPa at the other sample mats. The nanofibrous composite mats absorb less energy before fracturing as pristine PUUS. 

### 3.5. Static Contact Angle Determinations

Contact angle measurement was used to evaluate the wettability of the nanofibrous mats. Numerous studies have indicated that the surface wettability plays an important role in cell adhesion and proliferation, but this is equivocal [46,47]. This is because the mechanism of cell-biomaterial interaction is not so well understood, due to the complex factors, such as different surfaces properties like wettability, roughness, charge, cell types, protein adsorption and so on. For instance, some authors reported that the hydrophilic character of the biomaterial surfaces improve the cell interaction [48]. On the contrary, other researchers have shown that very hydrophobic surfaces lead to a good adhesion and proliferation of some cells, but they need more time to adapt to the surface [49,50]. In general, moderate wettability is more able to bind cells in comparison with highly hydrophilic or hydrophobic surfaces [51]. The reported contact angle measurements of our samples showed a contact angle of 88.61 for PUUS, 85.40 for PUUS-HPC and 84.83 for PUUS-βCD, respectively (Table 2). Hence, the nanofibrous composite mats showed slightly hydrophilic character than the pristine polyurethane mat. Thus, the decrease of the contact angle value is in accordance with the slight increase of the cell viability, as was observed in biological test reported below. 

### 3.6. Dynamic Vapour Sorption Analysis

**Figure 8:**
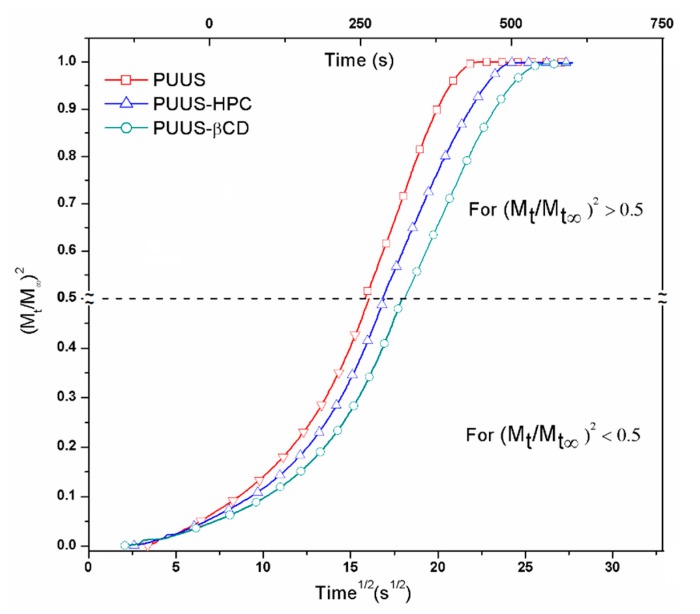
Diffusion plots for nanofibrous mat (PUUS) and nanofibrous composite mats (PUUS-HPC and PUUS-βCD).

The moisture sorption capacity can provide valuable information about materials because it is a critical factor in determination of their storage performance, stability, processing and application. This technique was used to determine the equilibrium curves and diffusion coefficients of the prepared samples, based on second Fick’s equation and the methods developed by Crank [52] and Balik [53]. As in a previous study [54], the diffusion coefficient (D) was calculated from the plot of normalized mass changing vs. time ½. The graphs are illustrated in Figure 8, for both short and long times. 

Therefore, at short times, the initial kinetics of sorption into the bulk may be described by the following equation:

(1) $$\frac{M_{t}}{M_{\infty }}=\frac{4}{l}\cdot \sqrt{\frac{D_{1}\cdot t}{\pi }}$$

where *D*_1_ is the diffusion coefficient when the values of *M*_t_/*M*_∞_ < 0.5 and l is the plate thickness. From Equation (1) results:

(MtM∞)2=16tD1πl2=K1t

where

K1=16D1πl2 and D1=K1πl216

At long times, when *M*_t_/*M*_∞_ > 0.5, the kinetics is described by the Equation (2):

(2) $$\frac{M_{t}}{M_{\infty }}=1-\frac{8}{\pi^{2}}\cdot e^{\frac{D_{2}\pi^{2}t}{l^{2}}}$$

where *D*_2_ is the diffusion coefficient when *M*_t_/*M*_∞_ > 0.5. From Equation (2) is obtained:

ln(1−MtM∞)=ln8π2−D2π2tl2=K2t

where

K2=−D2π2l2 and D2=−K2l2π2

Table 2 illustrates the diffusion coefficient values of the nanofibrous composite mats. It is observed that the diffusion coefficients for short or long time increased when HPC or β-CD were added. Thereby, the increase of the PUUS-HPC diffusion coefficient could be due to the increased volume structure of the HPC, which could lead to the high porosity of the matrix, involving vapor diffusion through pores. PUUS-βCD nanofibrous mats have the highest diffusion coefficient probably due to the toroidal shape of β-CD with secondary and primary hydroxyl groups from the glucose subunits. This structure increases the free volume between molecular chains, the composite structure is less packed and water molecules penetrate more easily inside [35]. Thus, the added compounds (HPC and β-CD) increase the number and size of cavities in the polymers, making diffusion easier. The higher increase of the diffusion coefficient is observed in the second part at *M*_t_/*M*_∞_ > 0.5, when the structures of the nanofibrous mats have enough time to expand and the diffusion process increase. 

### 3.7. DSC Analysis

**Figure 9:**
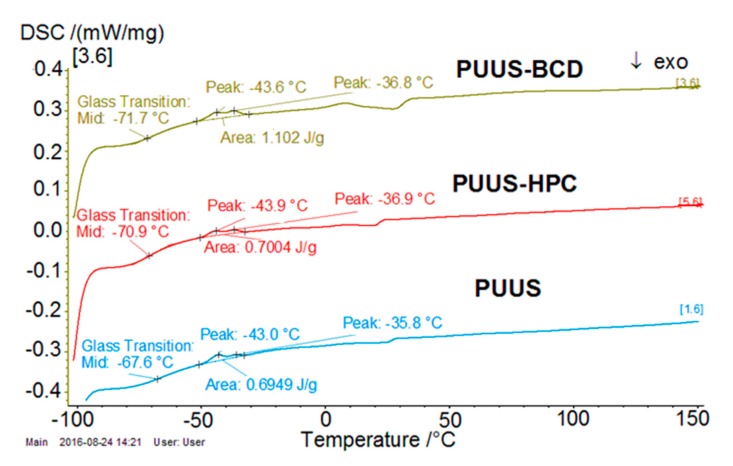
Differential scanning calorimetry (DSC) curves of nanofibrous mat (PUUS) and nanofibrous composite mats (PUUS-HPC and PUUS-βCD).

DSC is the most commonly used thermal method for the investigation of solid-state interactions between components. Thereby, the DSC profile of the nanofibrous mats is illustrated in Figure 9. DSC analysis in the range (−100)–(+150) °C reveals the presence of glass transition in the negative range, from −71.7 °C (PUUS-βCD) to −67.6 °C (PUUS), attributed to the PUUS component [36]. The glass transition temperature (*T*_g_) values highlighted by the DSC curves are centralized in Table 2. The incorporation of the HPC and β-CD into the polyurethane structures led to a slight decrease of the glass transition temperature values, acting as a plasticizer [43], as previously mentioned in mechanical characterization. The decrease is due to the complex structure of the inserted compounds (HPC and β-CD). For example, in the case of PUUS-βCD samples, the composite structure is influenced by the toroidal structure of β-CD that creates spaces between the polymeric chains, offering a greater mobility of them, which is able to slide past one another more easily. Some transitions are also visible in DSC curves at around −43 and −36 °C, respectively, corresponding to crystallization of polymeric structure, which are probably due to the reorganization of the polyurethane structures [55,56]. 

### 3.8. Biocompatibility Assessments

The preliminary biological analysis was performed in order to observe and analyze the interaction and response of the prepared nanofibrous mats in contact with cells, and also to identify their possible applications in the biomedical field. Therefore, two cell types were chosen—Human dermal fibroblast (HDF) and Human epidermoid cells (HEp2) cells. HDF cells are appropriate cell lines for use in cytotoxicity and tolerance tests, widely used as a model to mimic the interaction of materials with human skin, which take an active part in the immune response, inflammatory processes and wound healing [57,58]. HEp2 cells are widely used for the determination of the cytotoxic effects of different new materials [59,60]. The proper understanding of the interactions of these prepared materials with biological systems and their adverse effects is necessary for further development of some new matrices used for engineering scaffolds.

#### 3.8.1. In Vitro Cytotoxicity Tests

**Figure 10:**
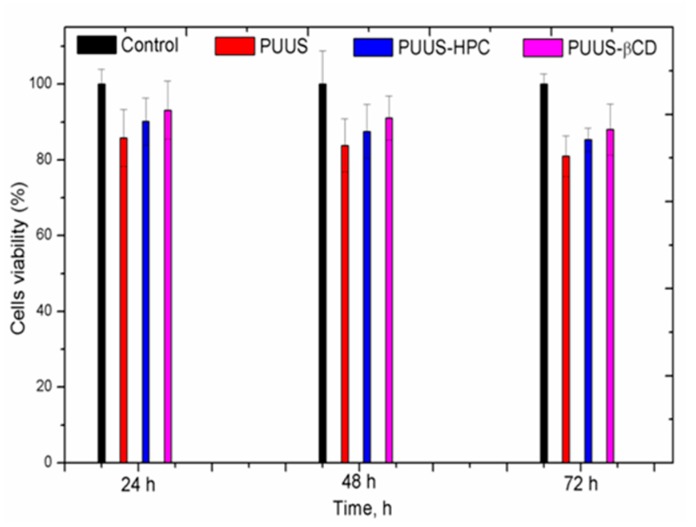
MTT assay of nanofibrous mat (PUUS) and nanofibrous composite mats (PUUS-HPC and PUUS-βCD).

The first cytotoxicity tests of the polyurethanes based nanofibrous composite mats were performed using HDF cells and yellow MTT assay. It is well-known from the literature [61] that this procedure of analyzing cell viability is fluently applied to assay toxicity, underlining the metabolic activity of cell cultures. Figure 10 shows the cell viability of the tested samples after 24, 48 and 72 h, respectively. Therefore, the cells cultured in contact with the pristine PUUS mat and composite mats (PUUS-HPC and PUUS-βCD) showed more than 80% of the cells viability. The prepared polyurethane based nanofibrous composite mats are non-cytotoxic for HDF cells and could certify their use in the medical field. 

Cell adhesion and proliferation is a complex phenomenon, not so well understood, and it is influenced by various properties such as: pore size [62,63], fiber diameters [30], wettability [48,50,51], roughness [64], surface chemistry [65], cell type and so on. Through the extensive literature data, it has been reported that the moderate wettability will promote the enhanced cellular response [51]. Our results show that the decrease of the contact angle values, previously reported, has led to a slight increase of the cell viability at the nanofibrous composite mats. 

#### 3.8.2. In Vitro Comparison of Cells Morphology and Cell Cycle Analysis

**Figure 11:**
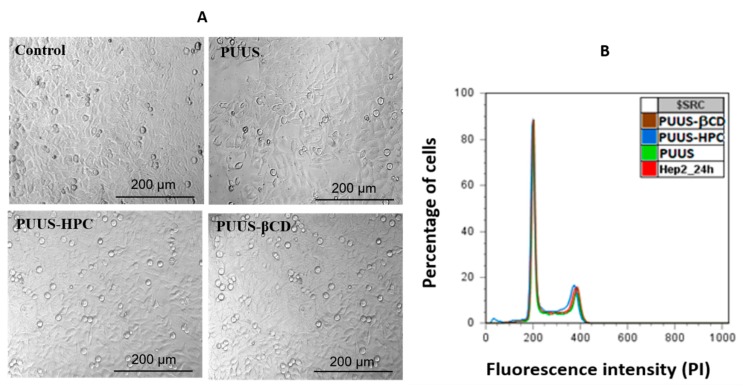
(**A**) Cell morphology of HEp2 cells on the nanofibrous mat (PUUS) and nanofibrous composite mats (PUUS-HPC and PUUS-βCD) surfaces; (**B**) cell cycle flow cytometry histograms.

Another cytotoxicity test was performed by using HEp2 cells, which are widely used for the determination of the cytotoxic effects of different materials. In order to evaluate the cell morphology, the polyurethanes based nanofibrous composite mats were incubated with HEp2 cells. The cell morphology for both polymer-bound cells and neighboring cells were analyzed in the same well and the images are illustrated in Figure 11A. The cells observation suggests that epithelial morphology is not affected by the presence of the polyurethane based nanofibrous composite mats. 

To analyze the effect harbored by the samples on the cell cycle, the cells were analyzed by flow cytometry. Figure 11B presents the influence of the mat samples on the cellular cycle of HEp2 (Y axis—intensity of fluorescence; X axis—the relative percentage number of cells). The cell cycle profile was determined by staining the DNA with a fluorescent dye (propidium iodide) and measuring its intensity. After 24 h, at 100 mg mL^−1^, the histograms are superimposed and no changes induced by PUUS, PUUS-HPC, PUUS-βCD nanofibrous mats, are observed compared to the HEp2 control.

#### 3.8.3. Microbial Adhesion Tests

**Figure 12:**
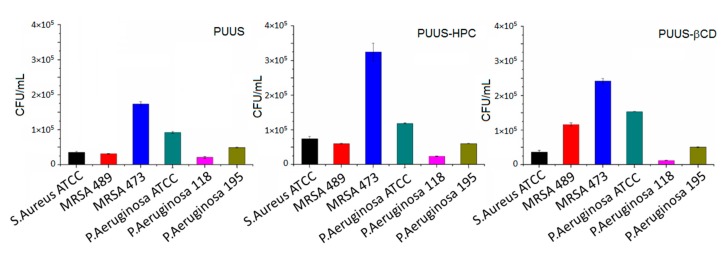
Different bacterial adhesion on nanofibrous mat (PUUS) and nanofibrous composite mats (PUUS-HPC and PUUS-βCD).

Adhesion of microbes, such as bacteria, fungi, protozoa, on surfaces causes multidrug-tolerant infections in humans and fouling of medical devices. It was found that the bacterial infections are the most common types of infections that cause worldwide morbidity [66]. The medical device is one of the sources of nosocomial infections. The attachment of the bacteria to the surface is a first step in the process of development of the infection and the physico-chemical properties of the surfaces play an important role in this attachment. Therefore, it is important to study the microbial adhesion on the biomaterial surfaces in order to improve their properties to resist against the bacterial adhesion for using further in the engineering of medical devices. Thus, the nanofibrous mats were incubated with different bacterial strains of *S. aureus* (ATCC, MRSA 489, MRSA 473) and *P. aeruginosa* (ATCC, 118, 195) and the results are illustrated in Figure 12. It is revealed that *S. aureus* strains showed higher adherence to the nanofibrous mats than *P. aeruginosa* strains, concluding that their effect on Gram-positive and Gram-negative bacteria is different, mainly due to their different cellular wall structure and also to the surface chemistry of the nanofibrous mats [67].

## 4. Conclusions

The goal of this research was to prepare nanofibrous composite mats based on polyurethane urea siloxane (PUUS), hydroxypropyl cellulose (HPC) and β-cyclodextrin (β-CD) and to understand the structure-properties relationship through investigation of the structural, morphological and biological properties. To achieve this purpose, solution (15% in DMF) of synthetized PUUS and PUUS blended with HPC or β-CD in a molar ratio of 9:1 (w/w), were eletrospun. The different analysis results showed that the properties of the nanofibrous composite mats were influenced by the addition of a small amount of HPC or β-CD. Therefore, in terms of surface morphology, depicted by SEM and AFM, the fibers are smooth, uniformly distributed, randomly oriented with the diameter at the nanoscale level ranged from 110 to 490 nm. A slight increase in fiber thickness at the composite mats is also observed. Mechanical tests reveal that the addition of the natural component (HPC and β-CD) affects more the tensile strength of the nanofibrous composite mats and less their ultimate elongation, acting as a plasticizer, leading also to a slight decrease of *T*g, as was observed in the DSC analysis. The nanofibrous composite mats showed a slightly hydrophilic character than the pristine polyurethane mat. The diffusion coefficients for short or long time increased when HPC or β-CD were added, due to the increased volume structure of the added component, which could lead to the high porosity of the matrix, involving vapor diffusion through pores. The cytotoxicity assay and microbial adhesion tests have good results in order to use these materials in the medical field. Thus, developed polyurethane based nanofibrous composite mats with enhanced physicochemical properties and biocompatibility showed great promise and potential for many biomedical applications, such as tissue engineering, wound dressing, immobilized enzymes and controlled drugs delivery. 

## Figures and Tables

**Table 1:** Experimental electrospinning conditions and mechanical properties of polyurethane based nanofibrous composite mats.

Sample		PUUS	PUUS-HPC	PUUS-βCD
Electrospinning condition	Voltage (kV)	15		
	Flow rate (mL h^−1^)	0.5		
	Needle size (gauge)	22		
	Spinning time (h)	10		
	Collector-needle distance (mm)	80		
Tensile strength (MPa)		4.14 ± 0.01	1.84 ± 0.008	1.32 ± 0.004
Elongation at break (%)		650 ± 17	580 ± 24	650 ± 13
Tensile modulus (MPa)		3.90 ± 0.01	0.87 ± 0.001	0.73 ± 0.002
Toughness (MPa)		11.57 ± 0.8	4.80 ± 0.5	4.16 ± 0.1

**Table 2:** Diffusion coefficients, contact angle values and glass transition temperatures resulted from the experimental data for polyurethane based nanofibrous composite mats.

Sample	* *K*_1_ × 10^3^ (s^−1^)	* *K*_2_ × 10^3^ (s^−1^)	*l* × 10^2^ (cm)	*D*_1_ × 10^6^ (cm^2^ s^−1^)	*D*_2_ × 10^6^ (cm^2^ s^−^^1^)	Contact Angle (°)	*T*_g_ (°C)
PUUS	0.804	−7.360	9	1.2781	6.04	88.61 ± 0.32	−67.6
PUUS-HPC	0.836	−16.38	9	1.3298	13.44	85.40 ± 0.12	−70.9
PUUS-βCD	1.393	−55.66	9	2.2149	45.68	84.83 ± 0.17	−71.7

* *K*_1_ is slope of linear regression between (*t* − *t*_R_) and (*M*_t_/*M*_∞_)^2^ for (*t* − *t*_R_) ≥ 0 and (*M*_t_/*M*_∞_)^2^ < 0.2; *t*_R_—time correlation for *M*_t_/*M*_∞_ = 0; *K*_2_ is slope of linear regression between *t* and ln (1 − *M*_t_/*M*_∞_) for −1.2 > ln > −3.0.
